# Long non-coding RNA Gas5 regulates proliferation and apoptosis in HCS-2/8 cells and growth plate chondrocytes by controlling FGF1 expression via miR-21 regulation

**DOI:** 10.1186/s12929-018-0424-6

**Published:** 2018-02-28

**Authors:** Xiong Liu, Yuqi She, Hongrong Wu, Da Zhong, Jian Zhang

**Affiliations:** 1 Department of Pathology, Xiangya Hospital, Central South University, Changsha, 410011 People’s Republic of China; 2 Department of Medical Administration, Children’s Hospital of Hunan Province, Changsha, 410011 People’s Republic of China; 3 Department of Orthopedic Surgery, Xiangya Hospital, Central South University, Changsha, 410011 People’s Republic of China; 4 Xiangya Hospital, Central South University, Xiangya Road No.87, Changsha, 410011 People’s Republic of China

**Keywords:** Gas5, miR-21, FGF1, Growth plate chondrocyte, Glucocorticoids

## Abstract

**Background:**

LncRNA Gas5 is known to be a key control element during growth, differentiation and development in mammalian species. However, the role and function of Gas5 in growth plate chondrocytes has not been determined.

**Methods:**

The overexpression and knockdown models of Gas5 and miR-21 in cells and animals were constructed. Cell survival was determined by MTT assay and flow cytometry. Animal biochemical indices were measured by enzyme-linked immunosorbent assay, hematoxylin/eosin staining, immunohistochemistry or *in situ* hybridisation. Dual luciferase reporter gene assay was carried out to study targeting.

**Results:**

First, we found the expression levels of fibroblast growth factor 1(FGF1) were up-regulated and miR-21 were down-regulated in Gas5 overexpressing model cells. Meanwhile, the expression levels of FGF1 and Gas5 were up-regulated in miR-21 knockdown model cells. Furthermore, cell proliferation was significantly promoted after Gas5 knockdown or miR-21 overexpression. Subsequently, Gas5 promoted apoptosis, while miR-21 suppressed apoptosis. Animal assays demonstrated that both Gas5 and dexamethasone suppressed proliferation and promoted apoptosis of growth plate chondrocytes, up-regulated FGF1 expression but reduced miR-21 expression. Finally, there was a binding relationship between Gas5, miR-21 and FGF1.

**Conclusion:**

We concluded that Gas5 regulated proliferation and apoptosis in growth plate by controlling FGF1 expression via miR-21 regulation.

**Electronic supplementary material:**

The online version of this article (10.1186/s12929-018-0424-6) contains supplementary material, which is available to authorized users.

## Background

Long non-coding RNAs (lncRNA) are non-protein coding RNA transcripts longer than 200 nucleotides [1]. Evidence suggests that numerous lncRNAs participate in diverse biological processes through gene-regulatory mechanisms, such as cell cycle control [2] and transcription [3]. However, knowledge concerning the roles of lncRNAs in bone formation and growth remains limited.

LncRNA growth-arrest-specific 5 (Gas5) has been reported to be a key control element during growth, differentiation and development in mammalian species [4]. Moreover, several studies have indicated that Gas5 acts as a tumour suppressor by regulating apoptosis appropriately [5], such as in breast cancer [6] and renal cell carcinoma [7]. Furthermore, other studies have reported that Gas5 binds to the DNA-binding domain of the glucocorticoid receptor (GR), thereby suppressing GR-induced transcriptional activity of endogenous glucocorticoid-responsive genes [8]. Meanwhile, several studies have revealed that glucocorticoids (GCs) affected growth plate and bone turnover, especially in children [9]. Therefore, we hypothesised that Gas5 may be involved in the formation and development of growth plate cartilage through regulating cell survival. Dexamethasone (Dex) is a type of glucocorticoid drug that can be used for anti-inflammation, antitoxic and anti-allergic treatments, and is widely used in clinic. However, the side effects of Dex can lead to more severe osteoporosis. Therefore, Dex was chosen as a representative of glucocorticoids used in this research.

MicroRNAs (miRNAs) are 18-27 nucleotide single-stranded non-coding RNA molecules involved in the negative regulation of gene expression through binding to complementary sequences in the 3-untranslated region (3′ UTR) of target mRNAs, thereby leading to either mRNA degradation or translational repression [10, 11]. MiR-21 is one of the earliest discovered miRNAs in human cells, and the expression of miR-21 is significantly up-regulated in various types of cancer [12]. MiRNA microarray analysis revealed that miR-21 expression in the Dex group was down-regulated six-fold compared with the non-Dex group in human bone marrow-derived mesenchymal stem cells (hBMSCs) [13]. Furthermore, Song J et al*.* showed that Gas5 is involved in the pathogenesis of osteoarthritis (OA) by acting as a negative regulator of miR-21, thereby regulating cell survival [14]. L Hu et al*.* also found that there is a target binding relationship between Gas5 and miR-21, and that Gas5 regulates migration and invasion of hepatocellular carcinoma (HCC) cells through the regulation miR-21 and its targets [15]. Therefore, we hypothesise that Gas5 may inhibit the formation and development of growth plate cartilage through the regulation of miR-21 and its targets.

## Methods

### Cell culture and treatment

The human chondrosarcoma-derived chondrocytic cell line HCS-2/8 was purchased from CELLBIO (Shanghai, China) with an identification report. Cells were cultured in Dulbecco’s Modified Eagle’s Medium containing 10% foetal bovine serum (Gibco Life Technologies, USA). HCS-2/8 cells were plated in 6-well plates and incubated overnight (37°C, 5% CO_2_), and then treated with Dex (0 μM, 50 μM, 150 μM, 450 μM) to induce the expression of the relevant factors.

### Real-time polymerase chain reaction (qRT-PCR) analysis

HCS-2/8 cells were harvested, and total RNA extracted using Trizol (Invitrogen, USA). Template cDNA was synthesised using a microRNA First Strand cDNA Synthesis Kit (Gene Copoeia, China) or Revert Aid First Strand cDNA Synthesis Kit (Fermentas, Canada). qRT-PCR detection was performed using a SYBR Green qRCR kit (Toyopo, Japan). *β*-actin and U6 were used as internal controls for lncRNA, mRNA and miR-21, respectively. The primers for miR-21 and U6 were purchased from (HmiRQP0316, HmiRQP9001, Gene Copoeia Inc. USA). GAS5 primer: sense, ATG GTT CTG CTC CTG GTA A, antisense, AGG TCT GCC TGC ATT TCT. FGF1 primer: sense, GTG GTG GAG GTA GGT ATT GG, antisense, GCA GCC TGT GAG TTT AGT TGT. Caspase 9 primer: sense, CTA CTT TCC CAG GTT TTG TTT CC, antisense, TCA CCG AAA CAG CAT TAG CG. *β*-actin primer: sense, AGG GGC CGG ACT CGT CAT ACT, antisense, GGC GGC ACC ACC ATG TAC CCT. The relative expression level was determined using the 2^-ΔΔCt^ method.

### Antibodies and reagents

Antibodies for western blotting and immunohistochemistry were: FGF1 polyclonal Antibody (ab9588, Abcam, USA, WB: 1:1000, IHC: 1:200), active caspase 9 polyclonal Antibody (ab32539, Abcam, WB: 1:1000, IHC: 1:200), Bcl-2 polyclonal Antibody (ab59348, Abcam, WB: 1:1000) and *β*-actin monoclonal Antibody (mAbcam8226, Abcam, WB: 1:500).

### Western blot

HCS-2/8 cells were harvested using RIPA buffer, and cell extracts were subjected to SDS-PAGE and immunoblotting, as previously described [16]. Briefly, active caspase 9, Bcl-2 and FGF1 expression in HCS-2/8 cells was determined using specific anti-active caspase 9, Bcl-2 and FGF1 antibodies, respectively.

### Establishment of Gas5 overexpression and Gas5 knockdown model

The Gas5 overexpression (pGMLV-6395) or knockdown (pGMLV-SC5) lentivirus were constructed. HCS-2/8 cells were transfected with the Gas5 overexpression or knockdown lentivirus and the negative control lentivirus, which included scramble sequences. The interference target was: AAG CCT AAC TCA AGC CAT TGG. G418 (400 μg/ml, Sigma, Germany) was used to select the cells stably overexpressing Gas5.

### Establishment of miR-21 overexpression and miR-21 knockdown model

The miR-21-mimics, miR-21-inhibitor and scramble controls were purchased from Gene Copoeia. The miR-21-mimics were used to activate miR-21 expression, while the miR-21-inhibitor was used to inhibit miR-21 expression. These complexes and their scramble negative controls (50 nM) were transfected in HCS-2/8 cells using Lipofectamine 2000 (Life Technologies) and cultured for the indicated time, following which the cells were harvested for assaying.

### Cell proliferation assay

Gas5 overexpression cells, Gas5 knockdown cells, miR-21-mimics cells and miR-21-inhibitor cells were seeded into 96-well plates and an 3(4,5-dimethylthiazol-2-yl)-2,5-diphenyltetrazolium bromide (MTT)-based assay was performed at the indicated times (0, 24, 48 and 72 h). The absorbance at 570 nm was used to determine the cell proliferation rates.

### Detection of cellular apoptosis and cycle assay

2×10^6^ Gas5 overexpression, miR-21-mimics, miR-21-inbitior or negative control cells were plated into 60 mm dishes and then treated with or without Dex for the indicated time. Cellular apoptosis was detected using the Annexin V-FITC Apoptosis Kit (BD Biosciences, USA) following the manufacturer’s instruction. The cell cycle was detected using PI (BD Biosciences).

### Ethics statement and rat models

We purchased 30 specific pathogen-free (SPF) male Sprague Dawley (SD) rats at 3-weeks-old from Shanghai SLAC Laboratory Animal Co. Ltd (China). Rats were housed under SPF conditions at 24±2°C and 55±5% relative humidity with a 12 h light-dark cycle. A commercial diet and potable water were available *ad libitum.* Rats were randomly divided into three groups (*n* = 10): the Gas5 overexpression group, the Dex treatment group and the control group. The Dex treatment group received Dex (200 μg/100 g, Sigma-Aldrich, Germany) through introperitoneal injection every day at 8:00 a.m., while the other group received 0.9% NaCl through introperitoneal injection. The Gas5 overexpression group received Gas5 overexpression lentivirus through intra-articular injection (1×10^9^ PFU), while the other group received the scrambled overexpression lentivirus through intra-articular injection.

### Body weight and body composition

Body weight was monitored every day at 8:00 a.m., the next day of the last time the rats were treated. The anatomy examination was performed under anaesthesia (10% chloral hydrate solution), while the tibiae and nose-tail lengths were measured three times by same operator. At the same time, serum was harvested for ELISA assay and tibiae tissues for tissue assay.

### ELISA

Rat Bone Alkaline Phosphatase (BALP) ELISA Kit (Kamiya, USA) was used to assess the expression of Bone Alkaline Phosphatase (BALP, one of the phenotypic markers of osteoblasts, which directly reflects the activity or functional status of osteoblasts) in serum from the rat models.

### Hematoxylin/eosin (HE) staining of tibiae tissue

Tibial sections were dewaxed and rehydrated, and then stained with HE following the instructions of HE Staining Kit (BBI, Canada).

### Immunohistochemistry

Tibiae sections were dewaxed and rehydrated, and then incubated overnight with the primary anti-FGF1 or active caspase 9 antibody at 4°C, and subsequently with an HRP-conjugated anti-rabbit-IgG for 1 h at room temperature. Colour development was performed using diaminobenzidine (DAB, Sangon, China). The sections were also counterstained with hematoxylin.

### *In situ* hybridisation

Tibiae sections were dewaxed, rehydrated, made transparent, ethanol-dehydrated V and then incubated overnight with biotin-tagged miR-21 probes. Colour development was performed using DAB. The sections were also counterstained with hematoxylin.

### Target prediction

MiR-21 target gene prediction was performed using the MiRanda software and online miRNA target prediction software (http://www.microrna.org/microrna/home.do).

### Dual luciferase reporter gene assay

For the luciferase reporter assay, the Dual Luciferase Reporter Gene Assay Kit (Promega, USA) was purchased and used following the manufacturer’s instructions. Briefly, FGF1-3′ UTR-psi-CHECK2 or Mut- FGF1-3′ UTR-psi-CHECK2 were used with miR-21-mimics or miR-21-inhibitor to co-transfect HCS-2/8 cells. Cells lysates were then assayed for luciferase using the Dual Luciferase Reporter Gene Assay system.

### Statistical analysis

In this study, all experiments were repeated at least three times. Quantitative data were expressed as mean ± standard deviation and analysed using SPSS 20.0 (IBM, USA). The difference between two groups was compared by the Independent Samples *t* test. Differences among three or more groups were compared by one-way ANOVA. *p* < 0.05 was considered to be statistically significant.

## Results

### The effect of Dex on HCS-2/8 cells

**Fig. 1:**
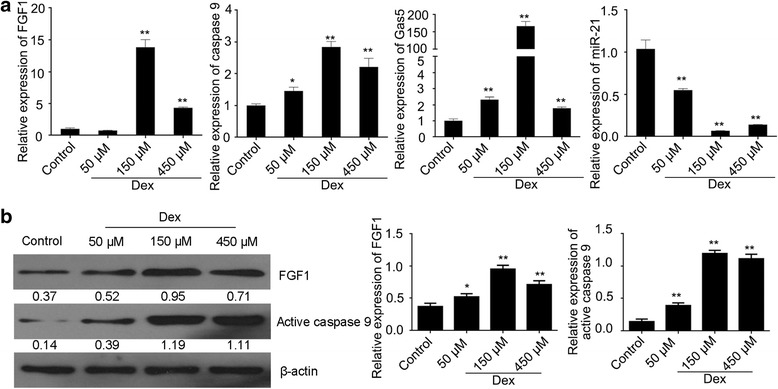
The relative expression changes in HCS-2/8 cells treated with Dex. **a** The expression of Gas5, miR-21, FGF1 and caspase 9 in HCS-2/8 cells treated with different doses of Dex as determined by qRT-PCR. **b** The expression of FGF1 and active caspase 9 proteins in HCS-2/8 cells treated with different doses of Dex as determined by western blot assays. Data are presented as mean ± s.d., *n* = 3, ^*^p < 0.05 or ^**^p < 0.01 *vs.* control group

HCS-2/8 cells were cultured in the presence of 50 μM, 150 μM or 450 μM Dex. The mRNA expression level of FGF1, caspase 9 and Gas5 were found to be up-regulated, while miR-21 was found to be down-regulated after Dex treatment. In particular, FGF1, caspase 9 and Gas5 expressions were significantly higher, while miR-21 expression was clearly lower at 150 μM Dex than other concentrations (Fig. 1a). The same changes were also observed at the FGF1 and active caspase 9 protein expression level (Fig. 1b). These data indicated that Dex induces cell apoptosis and influences FGF1, Gas5 and miR-21 expression. According to the data, 150 μM Dex treatments significantly influenced the expression of Gas5, miR-21, FGF1 and caspase 9 in HCS-2/8 cells, and this concentration was selected for further experiments.

### Gene expression changes in Gas5 and miR-21 function models in HCS-2/8 cells

**Fig. 2:**
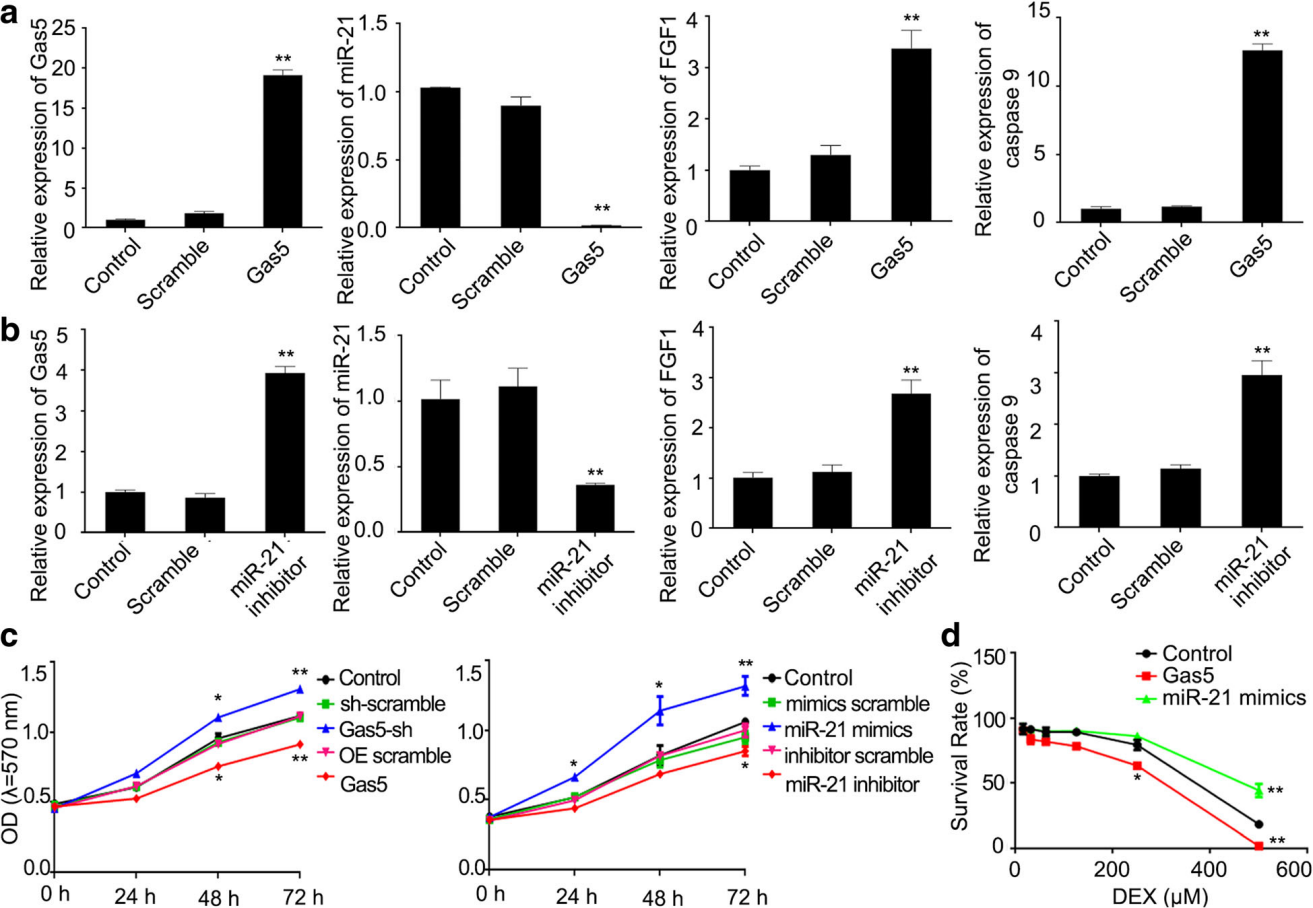
The effects of Gas5 and miR-21 in HCS-2/8 cells. **a** QRT-PCR assay was used to test the differential expression of Gas5, miR-21, FGF1 and caspase 9 in HCS-2/8 cells stably overexpressing Gas5. **b** The differential expression of Gas5, miR-21, FGF1 and caspase 9 in HCS-2/8 cells transfected with miR-21-inhibitor was determined by qRT-PCR assay. **c** Cell proliferation of HCS-2/8 gene function models assessed by MTT assays. **d** Cell survival rate assessed by MTT assays at 72 h. Data are presented as mean ± s.d., *n* = 3 or 5. ^*^p < 0.05 or ^**^p < 0.01 *vs.* control group

In order to explore the influence of Gas5 and miR-21 in HCS-2/8 cells, Gas5 overexpression and miR-21 knockdown cell models were constructed. In the Gas5 overexpression cell model, the expression of Gas5, caspase 9 and FGF1 was up-regulated, while miR-21 expression was down-regulated (Fig. 2a). These data indicated that the Gas5 overexpression cell model had been constructed successfully and that Gas5 was involved in the expression of miR-21, FGF1 and resulted in caspase 9-related cell apoptosis. For the miR-21 knockdown cell model, miR-21 expression was clearly decreased, while Gas5, FGF1 and caspase 9 were clearly increased (Fig. 2b). These data indicated that these cell models had been constructed successfully and that Gas5 may regulate miR-21 and FGF1 expression levels and resulted in caspase 9-related apoptosis in HCS-2/8 cells.

### Gas5 supresses cell proliferation and miR-21 promotes cell proliferation

As shown in Fig. 2c, cell proliferation was significantly promoted in the Gas5 knockdown and miR-21-mimics groups in HCS-2/8 cells, but was suppressed in the Gas5 overexpression and miR-21-inhibitor groups. These data indicated that Gas5 suppresses cell proliferation and miR-21 promotes cell proliferation. In addition, the combination of Dex with Gas5 overexpression and miR-21 inhibitor enhanced the killing effects on cell proliferation when compared with Dex treatment or Gas5 overexpression or miR-21 inhibitor alone (Fig. 2d). Dual luciferase assays in HCS-2/8 cells showed that there was a targeted relationship between Gas5 and miR-21 (Additional file 1).

### Regulation of Gas5 and miR-21 in cell apoptosis and cell cycle in HCS-2/8 cells

**Fig. 3:**
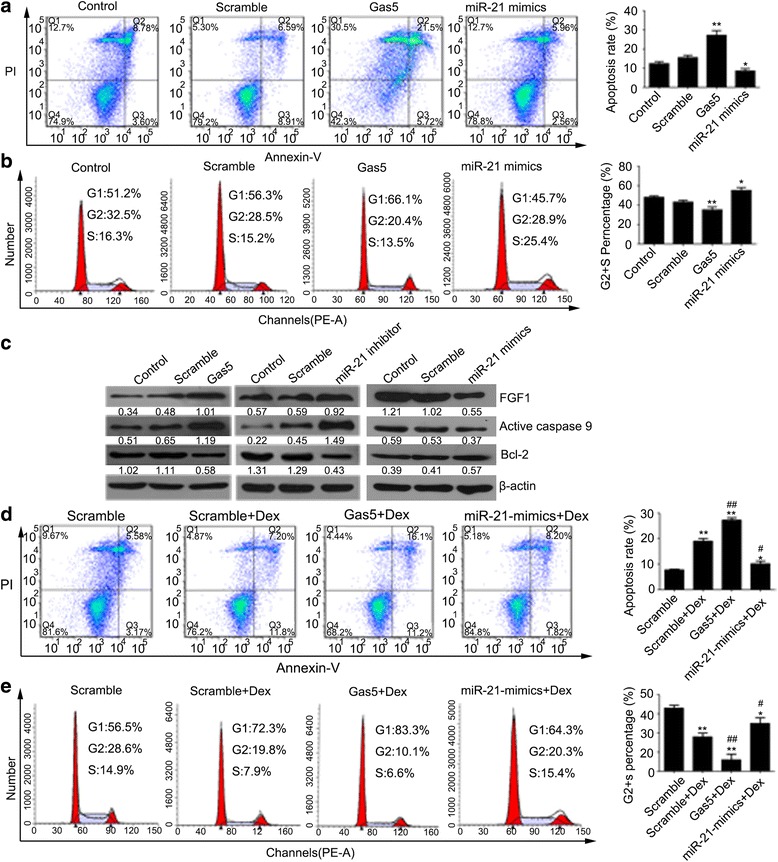
The regulation of Gas5 and miR-21 in cell apoptosis and cell cycle of HCS-2/8 cells treated with or without Dex. **a** Cell apoptosis analysis was quantified by Annexin V-FITC assay and fluorescence flow cytometry. **b** Cell cycle analysis was quantified by PI staining and fluorescence flow cytometry. **c** The expression of FGF1 and active caspase 9 proteins were assessed by western blot assays. **d** Cellular apoptosis analysis was quantified by Annexin V-FITC assay and fluorescence flow cytometry. **e** Cell cycle analysis was quantified by PI staining and fluorescence flow cytometry. Data are presented as mean ± s.d., n = 3. ^*^p < 0.05 or ^**^p < 0.01 *vs.* Scramble group. ^#^*p* < 0.05 or ^##^*p* < 0.01 *vs.* Scramble+Dex group

To confirm that Gas5 and miR-21 were involved in the regulation of cell apoptosis and the cell cycle, flow cytometry was performed using the Gas5 overexpression, miR-21-mimics and miR-21 inhibitor cell models. As shown in Fig. 3a and b, Gas5 overexpression inhibited cell cycle and promote cell apoptosis, while miR-21-mimics accelerated the cell cycle and inhibited cell apoptosis. Further studies in the Gas5 overexpression and miR-21 inhibitor cell models revealed up-regulated FGF1, active caspase 9 and down-regulated Bcl-2 protein levels were, while miR-21 mimics cell model revealed the opposite results (Fig. 3c). Considered together, it appeared that Gas5 overexpression promoted cell apoptosis in HCS-2/8 cells through the up-regulation of FGF1 and active caspase 9 but the down-regulation of Bcl-2, while miR-21 overexpression exhibited the opposite findings.

### Gas5 and miR-21 are involved in Dex-induced cell apoptosis and cell cycle

Flow cytometry was performed to determine the roles of Gas5 and miR-21 in apoptosis induced by Dex. As shown in Fig. 3, Gas5 overexpression enhanced the cell cycle inhibition and cell apoptosis induced by Dex (Fig. 3d), while miR-21-mimics exhibited the opposite findings (Fig. 3e).

### MiR-21 rescues the inhibition function of Gas5

**Fig. 4:**
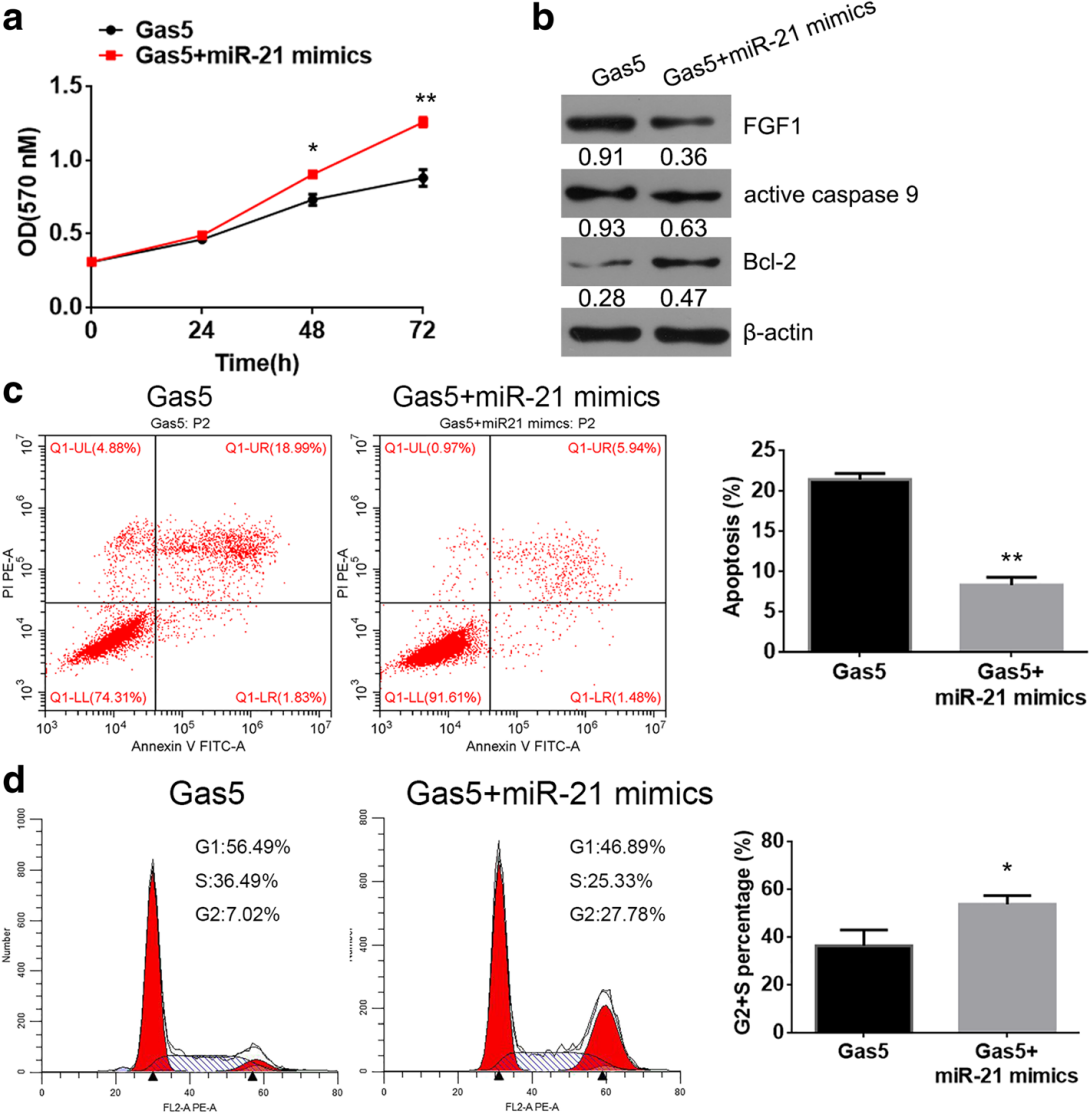
MiR-21 rescued the inhibition function of Gas5. **a** Cell proliferation of HCS-2/8 gene function models assessed by MTT assays. **b** The expression of FGF1 and active caspase 9 proteins were assessed by western blot assays. **c** Cell apoptosis analysis was quantified by Annexin V-FITC assay and fluorescence flow cytometry. **d** Cell cycle analysis was quantified by PI staining and fluorescence flow cytometry

From the results of the MTT assay, flow cytometry and western blotting, we established that the miR-21 mimics could reduce the inhibition function of Gas5 overexpression in HCS2/8 (Fig. 4).

### Gas5 inhibited the proliferation of growth plate chondrocytes in rats

**Fig. 5:**
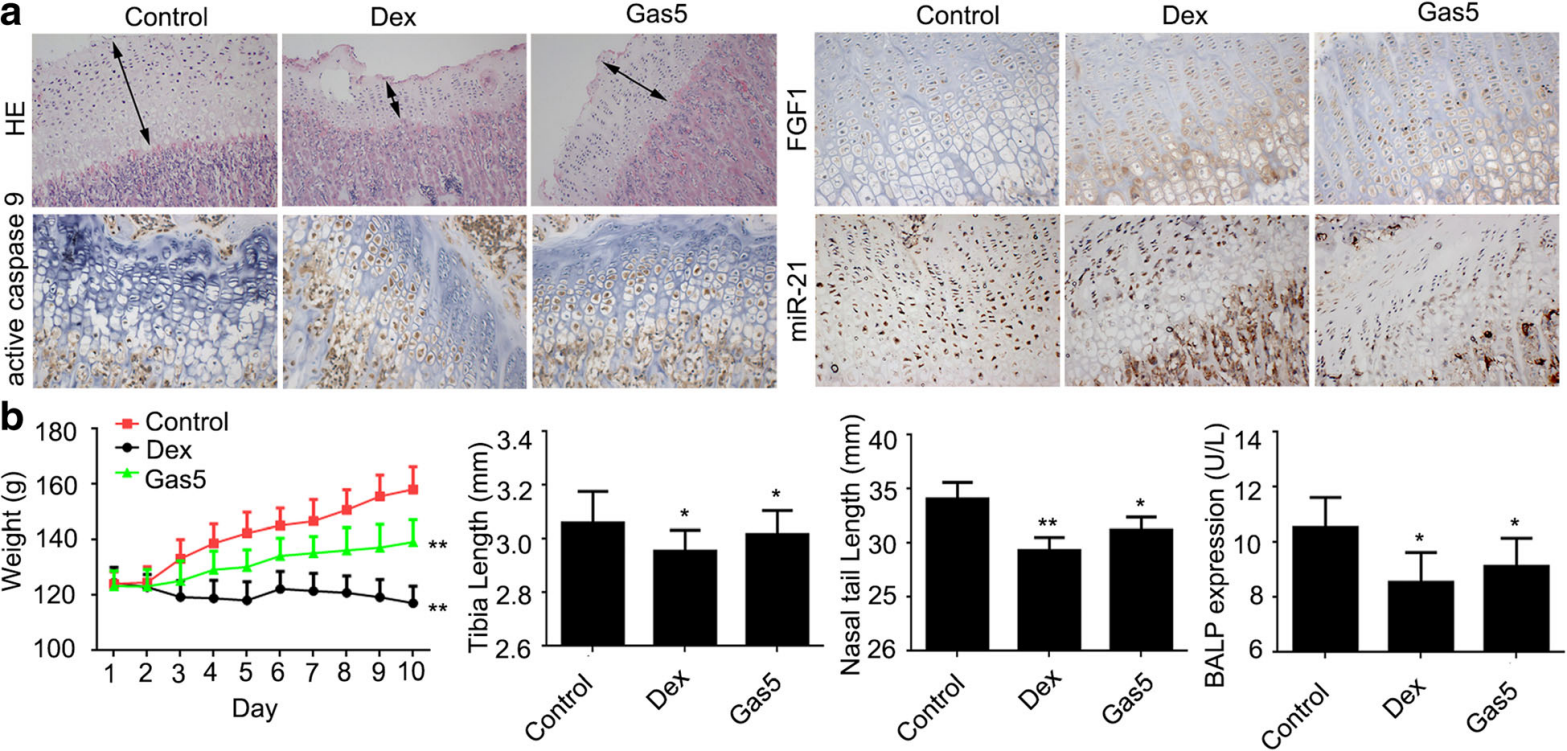
Rat model assay. **a** Rat tibiae growth plate sections from the control group, the Dex-treated group or the Gas5 overexpression lentivirus-infected group were prepared for staining analysis: HE staining (100 ×) for growth plate chondrocytes, IHC staining for FGF1 (400 ×) and active caspase 9 (400 ×), fluorescence *in situ* hybridisation for miR-21 (200 ×). **b** The weight, tibiae length, nose-tail length and BALP expression analysis of rats from the control group, the Dex-treated group or the Gas5 overexpression lentivirus-infected group. Data are presented as mean ± s.d., *n* = 10. ^*^p < 0.05 or ^**^p < 0.01 *vs.* control group. (**c**) GAS5 and FGF1 expressions in Dex-treated and untreated chondrocytes

To confirm that Gas5 and miR-21 were involved in proliferation and apoptosis in Dex-treated growth plate cartilage, rat models were established. Tibiae growth plate section staining showed that both Dex and Gas5 overexpression suppressed proliferation in growth plate chondrocyte, while up-regulating the expression of FGF1 and active caspase 9 and down-regulating the expression of miR-21 (Fig. 5a). The rat body composition assay showed that both Dex and Gas5 overexpression reduced the weight, tibiae length, nose-tail length and BALP expression (Fig. 5b). By analysing Dex-treated mouse chondrocyte expression chip data (GDS2802) in the GEO database, we obtained the expression of GAS5 and FGF1 in Dex-treated and untreated (DMSO) groups at 6 h. As shown in Fig. 5c, both GAS5 and FGF1 were up-regulated following Dex treatment for 6 h compared to the untreated group.

### FGF1 is a target of miR-21

**Fig. 6:**
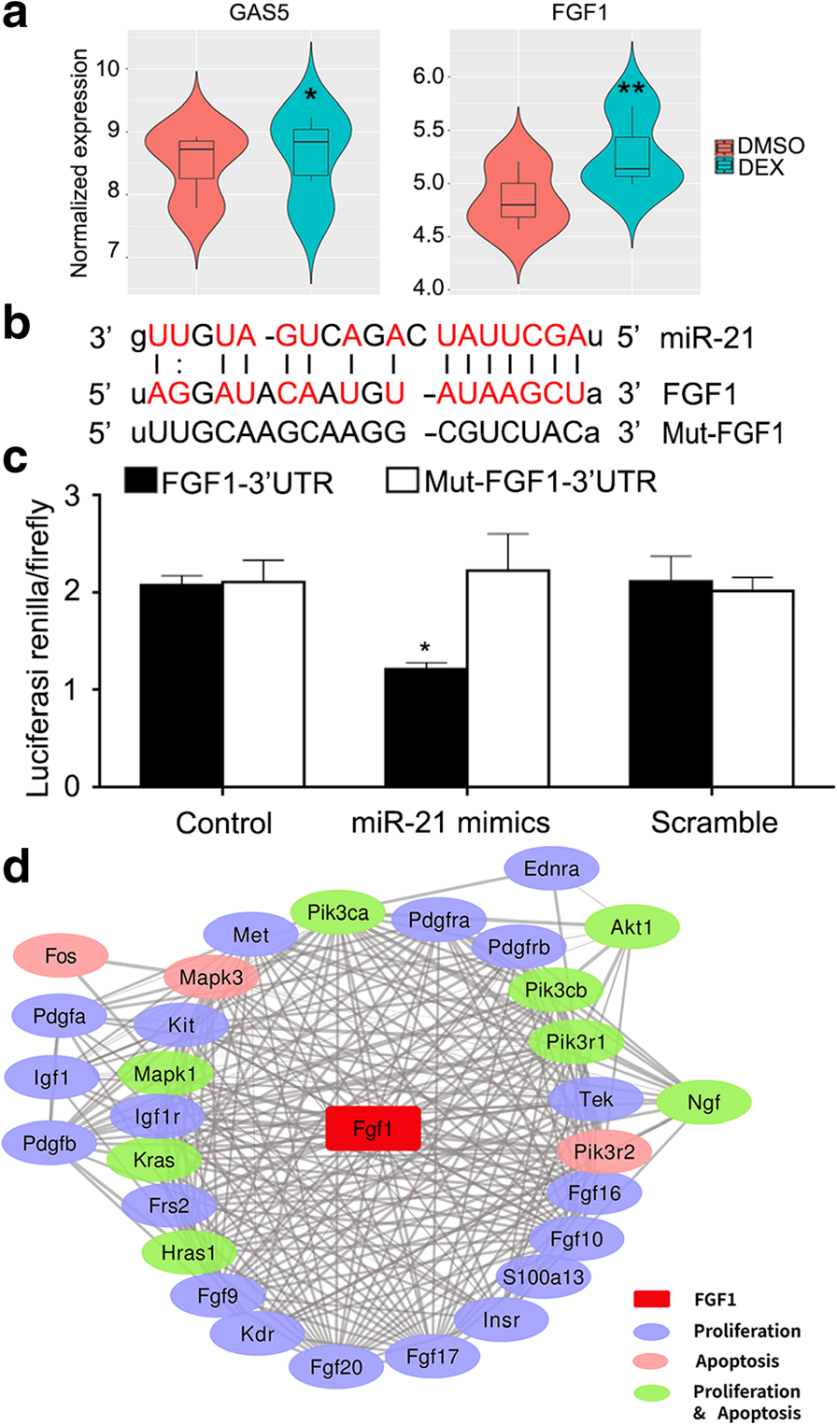
FGF1 is a target of miR-21. **a** Different expression levels of GAS5 and FGF1 between DEX treatment and no treatment mouse chondrocytes. *n* = 6. **b** Putative binding sites of miR-21 within FGF1. **c** Luciferase activities of miR-21 and FGF1 were measured and normalised according to renilla luciferase activity. Data are presented as mean ± s.d., *n* = 3. ^*^p < 0.05 or ^**^p < 0.01 *vs.* control group. **d** FGF1 and cell proliferation and apoptosis genes network diagram

Previous studies have shown that miR-21 is a target of Gas5 [15]. By analyzing DEX-treated mouse chondrocyte expression chip data (GDS2802) in the GEO database, we obtained the expression of GAS5 and FGF1 in DEX-treated and untreated (DMSO) groups. As shown in Fig. 6a, GAS5 and FGF1 increased in expression after DEX treatment. FGF1 happens to be a potential target of miR-21 by prediction using the online software (Fig. 6b). In the current study, dual luciferase reporter gene assay showed that FGF1 was indeed a target of miR-21 (Fig. 6c). To clarify the mechanism by which FGF1 affects proliferation and apoptosis, we collected genes related to apoptosis and cell proliferation (KEGG: Apoptosis pathway, GO: 0008283) through the KEGG and AmiGO databases. Among these genes, we screened for genes that interact with FGF1 through the STRING database to map the interaction of FGF1 with the genes involved in apoptosis and cell proliferation. In the figure, the gene surrounding the circle of FGF1 is a gene that directly interacts with FGF1, while the other edge genes are important genes that interact with FGF1 secondary. It can be seen from the figure and HRAS, KRAS, MAPK3 and other cell proliferation and apoptosis process of key genes have a direct interaction (Fig. 6d).

## Discussion

This report provides the first demonstration that Gas5 regulates proliferation and apoptosis in growth plate chondrocytes via suppression of FGF1 targeting by miR-21.

Based on the hypothesis that Gas5 contributes to the formation and development of growth plate cartilage through the regulation of miR-21 and its targets, dexamethasone (Dex), which is known to be a steroid with the comparative biological potency of glucocorticoids (GCs) and which is widely used in children and adults with allergic disorders, skin conditions, ulcerative colitis, arthritis, lupus, psoriasis, or breathing disorders, was selected for use in this study [17]. Previously studies have shown that Dex induces apoptosis and reduces cell proliferation in growth plate chondrocytes [16]. Moreover, caspases and the akt-phosphatidylinositol 3’-kinase signalling pathway contribute to Dex-induced apoptosis in proliferative chondrocytes [18]. At the start of our study, HCS-2/8 cells were treated with different doses of Dex, and a significant difference in Gas5, miR-21, FGF1 and active caspase 9 expression was observed at a Dex concentration 150 μM. We also hypothesised that Gas5, miR-21, FGF1 and caspase 9 play critical roles in proliferation and apoptosis in growth plate chondrocytes.

In order to prove our hypothesis, overexpression and knockdown models of Gas5 and miR-21 were established, and we found that Gas5 contributed to pro-apoptosis by increasing active caspase 9 and FGF1 expression but decreasing miR-21 expression. In contrast, miR-21 contributes to pro-proliferation by acting as a negative regulator of Gas5, FGF1 and active caspase 9.

Previous studies have shown that the expression of Gas5 is negatively regulated by mTOR [19], which promotes cell proliferation. In the present study, Gas5 overexpression down-regulated miR-21 levels and miR-21 negatively regulated Gas5 levels, suggesting the interaction between Gas5 and miR-21. Our studies also revealed that elevated levels of Gas5 promoted apoptosis and reduced the cell cycle in HCS-2/8 cells and enhanced apoptosis induction by Dex, which is consistent with previous research that showed Gas5 to be a ‘riborepressor’ of the glucocorticoid receptor and to promote cell apoptosis [9]. In addition, rat model assays in the current study also revealed that Gas5 suppressed proliferation and promoted apoptosis of growth plate chondrocytes. Moreover, Gas5 levels are also significantly reduced in many types of cancer cells [6, 7] and increasingly, studies on Gas5 as a tumour suppressor are being carried out in the cancer field [5]. Thus, we speculate that it may represent a novel target for diagnosis and treatment in relation to diseases in growth plate.

Emerging evidence has shown that miR-21 takes part in bone remodelling via the promotion of proliferation and anti-apoptosis effects [20]. In line with these findings, our results identified that miR-21 promoted proliferation and accelerated the cell cycle in HCS-2/8 cells and weakened apoptosis induction by Dex. Interestingly, miR-21 levels increased in tibiae tissue treated with Dex or infected with Gas5 overexpression lentivirus, and decreased in HCS-2/8 cell treated with Dex (150 μM) or in stably Gas5 overexpression cell lines. One reason for these results may be the dose of Dex treatment; however, other reasons may be the complex interplay in organisms. For instance, miR-21 negatively regulates Gas5 or/and downstream protein factors.

FGF is a member of the FGF family, which controls broad mitogenic and cell survival activities [21]. Chen et al. demonstrated that the Ser252Trp mutation in FGF receptor 2 was vital in FGF signalling and reduced the proliferation of BMSCs [22]. In the present study, FGF1 mRNA and protein levels were up-regulated in HCS-2/8 cells treated with Dex, and in the Gas5 overexpression or miR21-inhibition models, while FGF1 protein level was up-regulated in tibiae tissue treated with Dex or infected with Gas5 overexpression lentivirus. Together, these results suggested that FGF1 played a key role in apoptosis induced by Gas5 or miR-21 in growth plate cartilage. Further studies conclusively showed that FGF1 was a target protein of miR-21 according to the dual luciferase reporter gene assay. Moreover, previous reports revealed that Gas5 targets miR-21 and regulates cell survival. Thus, our study indicates that Gas5 contributes to the formation and development of growth plate cartilage through the regulation of miR-21 and its targets.

Apoptosis, a form of programmed cell death, plays a central role in tissue homeostasis, and deregulation of apoptosis has been implicated in numerous pathological conditions [23]. Caspase 9 is a member of caspase family of cysteine proteases that have been implicated in apoptosis and cytokine processing [24], whereas Bcl-2 is specifically considered an important anti-apoptotic protein but is not considered a proto-oncogene as it is not a growth signal transducer [25]. In the current study, the expression of active caspase 9 was increased and Bcl-2 was decreased according to qRT-PCR and western blot assays. These results further prove that Gas5 and miR-21 are involved growth plate cartilage apoptosis.

## Conclusions

It was concluded in the present study that Gas5 regulated proliferation and apoptosis in growth plate chondrocytes by controlling the expression level of FGF1 via miR-21 regulation. The findings of the current study provide a promising insight into the molecular mechanism and target therapy of growth retardation.

## Additional file

Additional file 1: GAS5 is a target of miR-21. (A) Putative binding sites of miR-21 within GAS5. (B) Luciferase activities of miR-21 and GAS5 were measured and normalised according to renilla luciferase activity. Data are presented as mean ± s.d., *n* = 3. ^*^p < 0.05 *vs.* control group. (TIFF 1994 kb)
