# Supplementary figures and images for: Long non-coding RNA Gas5 regulates proliferation and apoptosis in HCS-2/8 cells and growth plate chondrocytes by controlling FGF1 expression via miR-21 regulation
Source: J Biomed Sci. 2018 Feb 28;25:18. doi: 10.1186/s12929-018-0424-6 (PMC5830091; doi:10.1186/s12929-018-0424-6)

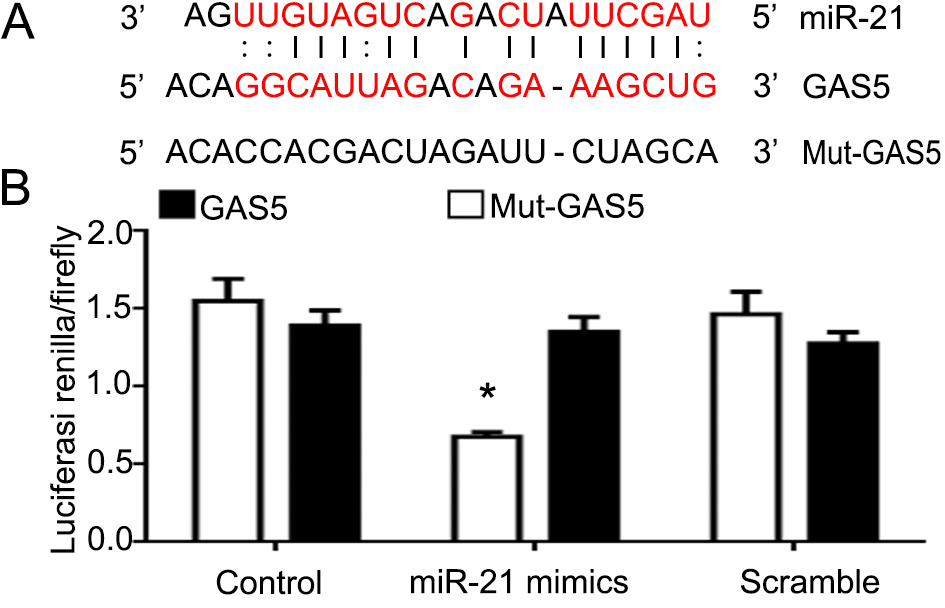

Supplement: Supplementary file 1 — GAS5 is a target of miR-21. (A) Putative binding sites of miR-21 within GAS5. (B) Luciferase activities of miR-21 and GAS5 were measured and normalised according to renilla luciferase activity. Data are presented as mean ± s.d., n = 3. *p < 0.05 vs. control group. (TIFF 1994 kb) [file 12929_2018_424_MOESM1_ESM.tif]
